# Recent Advances in Structuring and Patterning Silicon Nanowire Arrays for Engineering Light Absorption in Three Dimensions

**DOI:** 10.1021/acsaem.1c02683

**Published:** 2021-10-28

**Authors:** Theresa Bartschmid, Fedja J. Wendisch, Amin Farhadi, Gilles R. Bourret

**Affiliations:** † Department of Chemistry and Physics of Materials, University of Salzburg, Jakob Haringer Strasse 2A, A-5020 Salzburg, Austria; ‡ Nanoinstitut München, Department of Physics, Ludwig-Maximilians-University Munich, 80539 München, Germany

**Keywords:** Si nanowire, array, metal-assisted chemical etching, lithography, patterning, 3DEAL, light absorption

## Abstract

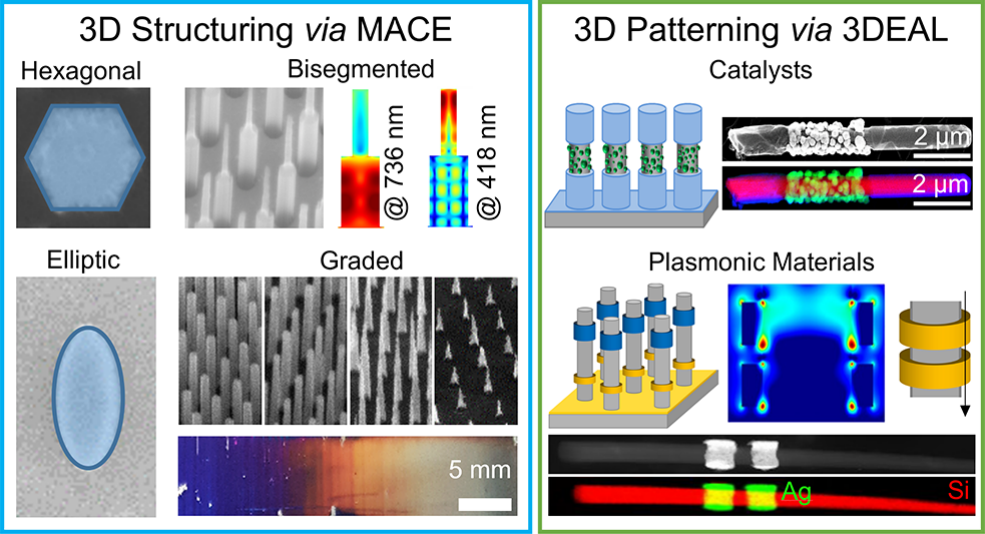

Vertically aligned silicon nanowire (VA-SiNW) arrays can significantly enhance light absorption and reduce light reflection for efficient light trapping. VA-SiNW arrays thus have the potential to improve solar cell design by providing reduced front-face reflection while allowing the fabrication of thin, flexible, and efficient silicon-based solar cells by lowering the required amount of silicon. Because their interaction with light is highly dependent on the array geometry, the ability to control the array morphology, functionality, and dimension offers many opportunities. Herein, after a short discussion about the remarkable optical properties of SiNW arrays, we report on our recent progress in using chemical and electrochemical methods to structure and pattern SiNW arrays in three dimensions, providing substrates with spatially controlled optical properties. Our approach is based on metal-assisted chemical etching (MACE) and three-dimensional electrochemical axial lithography (3DEAL), which are both affordable and large-scale wet-chemical methods that can provide a spatial resolution all the way down to the sub-5 nm range.

## 1 Introduction

Vertically aligned silicon nanowire (VA-SiNW) arrays can significantly trap light thanks to various enhanced absorption and scattering processes. As such, they are a promising platform to improve solar cell design by (i) reducing front-face reflection; (ii) allowing the fabrication of thin, flexible, and efficient silicon-based solar cells; and (iii) potentially decreasing fabrication cost by lowering the amount of silicon required to absorb the incoming solar light. Because the strong interaction of SiNW arrays with light is highly dependent on the array geometry,^1−13^ the development of synthetic methods to finely tune the array dimension and morphology is necessary. Additionally, the controlled structuring and patterning of Si in three dimensions can provide an additional degree of freedom by providing the opportunity to control light absorption in three dimensions, which could be beneficial for improved photovoltaic and photocatalytic systems. After discussing the remarkable optical properties of SiNW arrays, we report on our recent progress in using chemical and electrochemical methods to structure and pattern SiNW arrays in three dimensions, which provide substrates with spatially controlled optical properties at both the nanoscale and the macroscale.

## 2 Optoelectronic Properties of Silicon Nanowires

**Figure 1:**
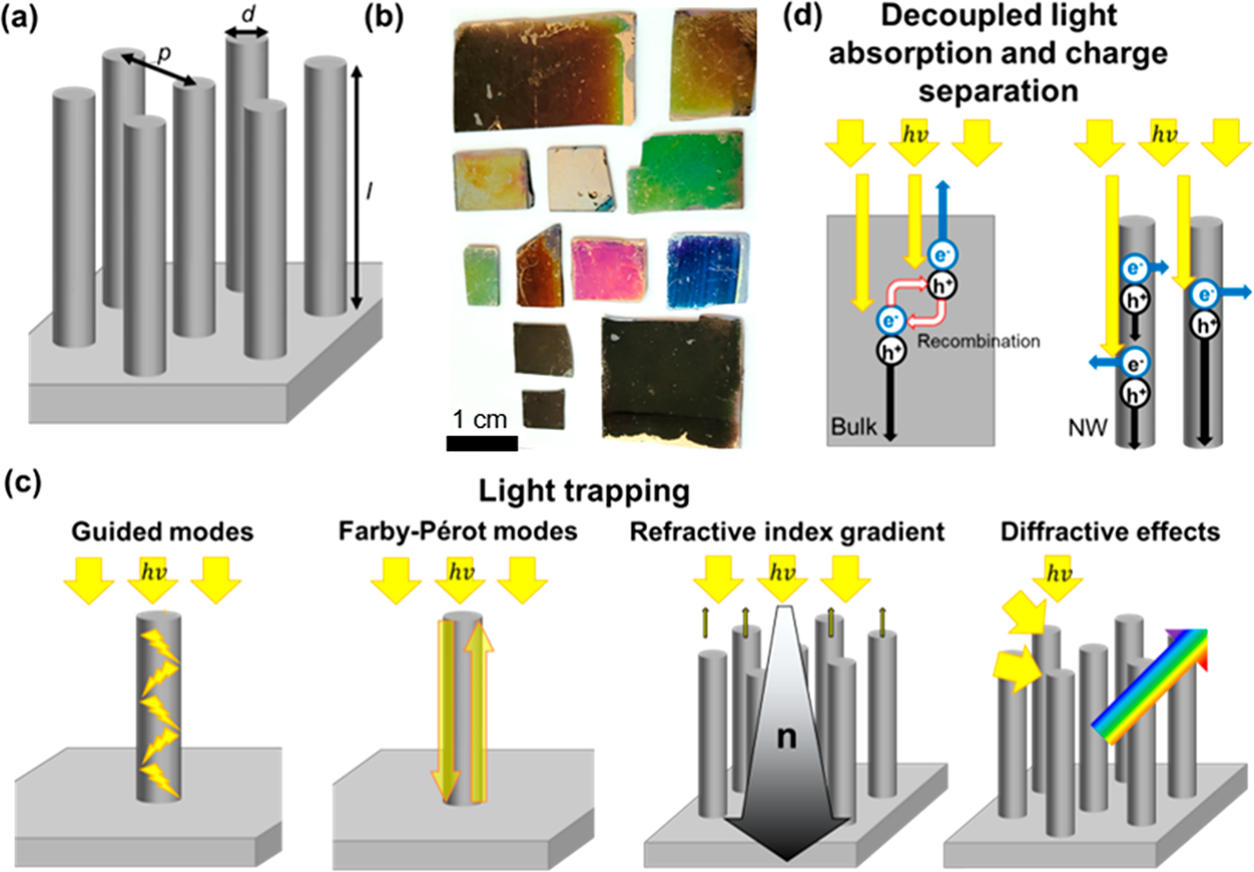
Optoelectronic properties of SiNW arrays. (a) Scheme showing the geometrical parameters that can be easily adjusted during the synthesis of SiNW arrays: wire diameter (*d*), length (*l*), and array pitch (*p*). (b) Photographs of various SiNW arrays synthesized in our laboratories showing colors that cover the whole visible spectrum, including black silicon surfaces that significantly trap the incident light. (c, d) Schematic illustration of (c) various light trapping effects that can lead to enhanced absorption, reduced reflection, and diffraction; and (d) decoupled light absorption from the charge separation process that can lead to reduced charge recombination.

The rich interaction of VA-SiNW arrays with light (Figure 1a,b) gives rise to a variety of optical effects (Figure 1c) that include waveguiding,^8−11^ Fabry–Pérot resonances,^9,12^ low reflectivity (*moth-eye* effect),^1−7^ diffractive effects,^12^ and near-field coupling.^13^ Thus, these arrays have highly tunable light reflection, absorption, and scattering properties, while providing the opportunity to decouple the light absorption from the charge separation process, which minimizes charge recombination (Figure 1d). In the following, the different interaction mechanisms of light with the SiNW arrays are briefly explained. For a more detailed description, we direct the reader to the articles and review papers cited in this section.

### 2.1 Leaky Waveguide Modes

**Figure 2:**
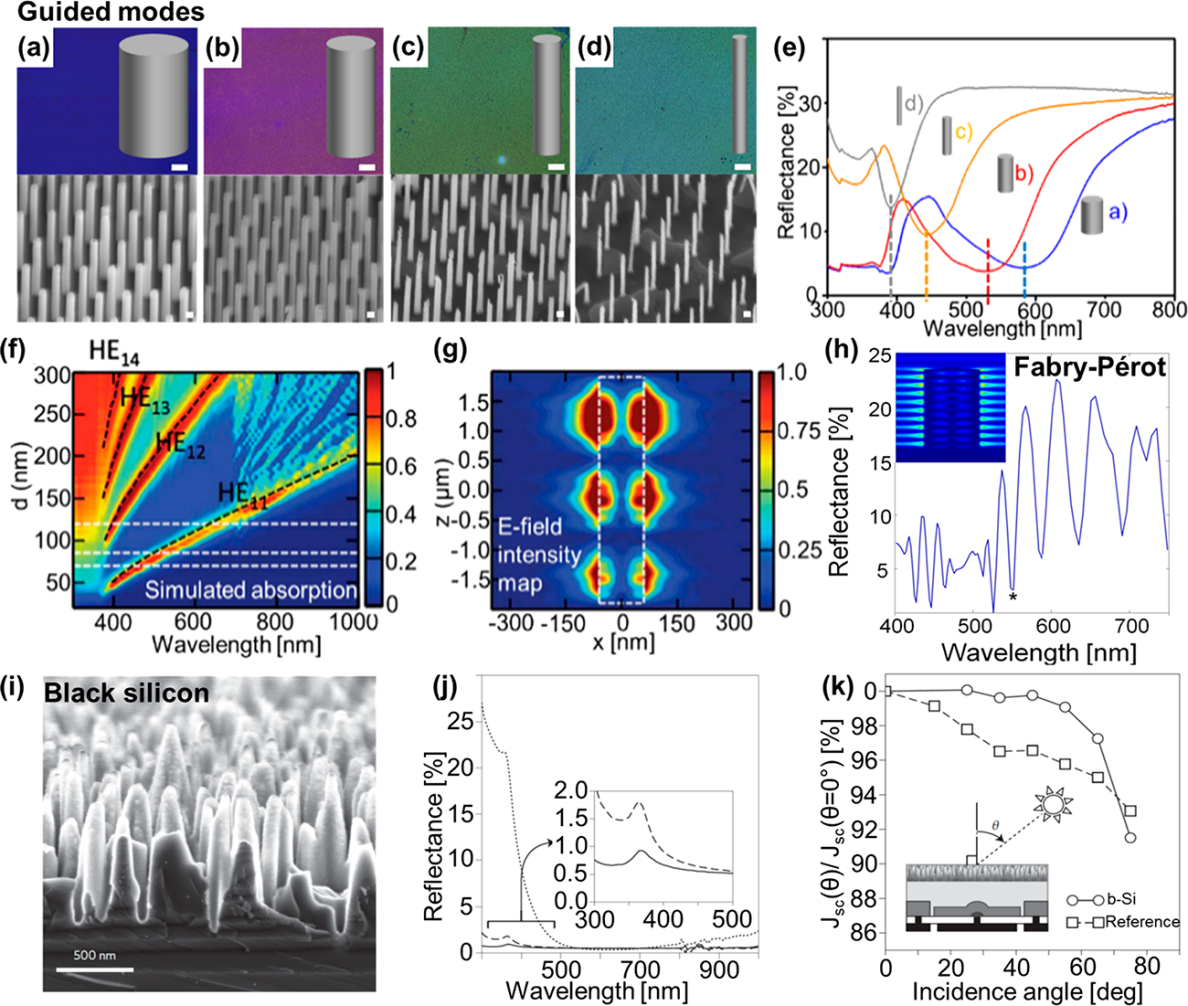
Guided modes, Fabry–Pérot resonances, and black silicon for solar cells. (a–g) Guided modes. (a–d) Top row, optical microscopy images (scale bars: 100 μm); bottom row, corresponding secondary electron SEM images tilted images (scale bars: 100 nm) of various SiNW arrays (*p* = 430 nm, *l* ∼ 1700 nm). (a) *d* = 118 nm. (b) *d* = 87 nm. (c) *d* = 77 nm. (d) *d* = 63 nm. (e) Reflectance spectra of the SiNW arrays shown in panels a–d. Adapted from ref (11). Copyright 2020 American Chemical Society. (f) Contour plot of simulated absorption as a function of wavelength and nanowire diameter (*p* = 800 nm, *l* = 3800 nm). Dotted black lines indicate different waveguiding modes. (g) Electric field intensity maps of leaky mode resonances for *d* = 120 nm at λ = 670 nm (HE_11_ mode). The dotted white line indicates the edge of the nanowire cross-section. (f, g) Reprinted with permission from ref (10). Copyright 2012 The Optical Society. (h) Reflectance spectrum of VA-SiNW array showing oscillations characteristic of Fabry–Pérot resonances (*d =* 200 nm, *l =* 2.4 μm). Inset: Corresponding normalized magnitude of the electric field at the wavelength noted with an asterisk. Modified from ref (12). Copyright 2016 American Chemical Society. (i–k) Black silicon for solar cells. (i) SEM image of black silicon prepared via reactive ion etching. (j) Reflectance spectra of a bare black-Si sample (dashed line), a black-Si sample coated with 20 nm of Al_2_O_3_ (black solid line), and typical random silicon pyramids coated with 90-nm-thick Al_2_O_3_ film (black dotted line). A dramatic decrease in reflectance is observed for black silicon, especially below 500 nm. (k) Relative photocurrent, with respect to the photocurrent at normal incidence, for different light incidence angles for both the black-Si (circles) and reference (squares) solar cells. The light incidence angle θ is defined in the inset. Adapted by permission from ref (14). Copyright 2015 Springer Nature.

Semiconductor nanowires can act as subwavelength dielectric cylindrical waveguides, where the light is guided and trapped inside the wire, causing a higher light absorption at specific wavelengths, responsible for the vivid colors of SiNW arrays with wire diameters *d* in the sub-300 nm range (Figure 2a–d).^8−11^ Characteristic dips are seen in the reflectance spectra of such VA-SiNW arrays (Figure 2e). Leu et al. studied the diameter-dependent waveguiding behavior of SiNWs with diameters ranging from 25 to 300 nm (pitch, *p* = 700 nm, and length, *l* = 3800 nm).^10^ A contour plot of the simulated absorption, seen in Figure 2f, shows that SiNWs can sustain so-called hybrid HE_1n_ modes (black dotted lines), where 1 is the azimuthal mode number and *n* the radial mode number, which represents the radial variation of the electromagnetic field.^9^ All modes red-shift with increasing diameters, while larger nanowires can sustain multiple modes: Light absorption can be increased at different wavelengths. The simulated electric field (E-field) intensity map (Figure 2g) shows that the HE_11_ mode is enhancing the E-field not only inside but also periodically around the nanowires, leading to so-called leaky waveguiding. Additionally, under normal incidence, the leaky waveguide modes can couple at a short pitch.^9^ This can lead to a red shift and broadening of the resonance, while the enhanced E-fields generated around the nanowires can confine the light inside the gap region, increasing further the E-field enhancement. Such a near-field coupling has been used to enhance molecular sensing on silicon nanowires.^13^

### 2.2 Fabry–Pérot Resonances

VA-SiNWs irradiated under normal incidence (i.e., parallel to the NW long-axis) can sustain Fabry–Pérot resonances where the incident light is reflected at the bottom and the top of the nanowire array to form a standing wave, leading to a characteristic dip in the reflectance spectrum.^9,12^ Such Fabry–Pérot cavities are observed whenever the round trip nanowire length 2*l = m*λ_eff_, where *m* is an integer, and λ_eff_ is the effective wavelength in the nanowire array, defined by λ_eff_ = λ_0_/*n*_eff_, where λ_0_ is the wavelength of light in vacuum and *n*_eff_ is the effective refractive index. Such a condition can be met at several wavelengths for large nanowire lengths, seen by oscillations in the reflectance spectrum of such VA-SiNW arrays (Figure 2h). The Fabry–Pérot modes can be easily identified by the evenly spaced nodes of the E-field along the NW long-axis, seen on the E-field maps calculated via numerical simulations (inset Figure 2h). Additionally, the position of these resonances depends on the NW length.^11^

### 2.3 Low Reflectivity Due to a Refractive Index Gradient (Moth-Eye Effect, Black Silicon)

Because of the high silicon’s refractive index, untreated silicon solar cells reflect a considerable amount of the incoming solar light (i.e., >30% in the UV to near-IR range). This is because light reflection occurs and is highest when a sharp and large change in refractive index occurs between two interfaces. The use of nanostructured surfaces such as silicon nanowire arrays can gradually change the refractive index, leading to a significant reduction of the substrate reflectivity thanks to the so-called *moth-eye* effect.^2−7^ Such black silicon can almost completely suppress light reflection, affording the preparation of Si substrates with reflectivity values down to 1–2% across the UV to near-IR range.^1,14−16^ This dramatic change of the silicon surface optical property has already shown potential for increasing the conversion efficiency of solar cells across a wide range of incidence angles (Figure 2i–k).^1,14−16^ Thus, black silicon can lower the amount of active material while significantly reducing light reflection and has potential for fabricating high-efficiency and low-cost thin film solar conversion systems.

### 2.4 Diffractive Effects

Periodical arrays of nanowires can show diffractive effects. Depending on the diffraction order, wavelength, incidence angle, and array pitch, vivid structural colors can be observed. For 2D hexagonal lattice photonic crystals, the first order diffraction occurs for , where λ is the position-dependent peak diffracted wavelength and θ the incidence angle.^12^

### 2.5 Decoupled Light Absorption and Charge Separation

In addition to their superior optical properties in comparison to those of bulk silicon, SiNWs benefit from their one-dimensional geometry which can decouple light absorption from the charge separation process (Figure 1d).^17−19^ In a photovoltaic or photocatalytic device, the recombination of the photoexcited charge carriers before their extraction can reduce conversion efficiencies. For bulk material with a planar surface, the minority charge carrier diffusion length *L*_d_ should be larger than the semiconductor thickness *t*, which should be large enough to ensure sufficient light absorption. For a bulk material, this can lead to either strong recombination if *t* is too large (i.e., *L*_d_ < *t*) or improper light absorption if *t* is too small.^7^ Within nanowires, charge separation can occur in the radial direction, orthogonal to light absorption, which occurs along the wire axis. This contributes to lower charge recombination within nanowire devices and allows the use of low-purity semiconductors with small *L*_d_, potentially reducing fabrication costs.^17,20,21^

## 3 Previous Attempts at Structuring and Patterning SiNWs

### 3.1 Structuring via Selective Etching

**Figure 3:**
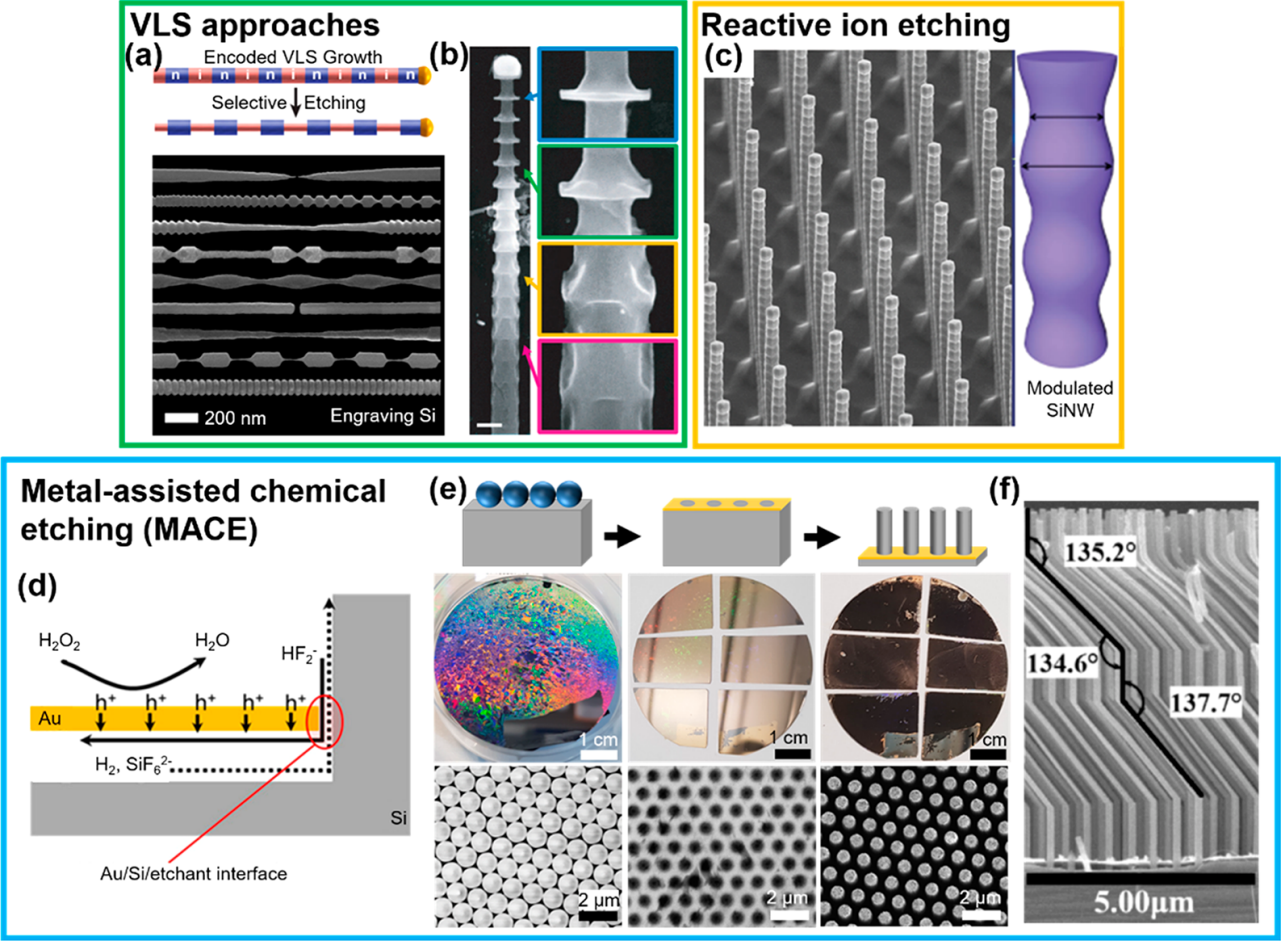
Structuring SiNWs via selective etching. (a, b) Modified VLS synthesis in combination with selective etching. The structures are etched anisotropically due to incorporated (a) acceptor/donor atoms (adapted from ref (23); copyright 2013 American Chemical Society) or (b) gold impurities (adapted with permission from ref (22); copyright 2015 The American Association for the Advancement of Science). (c) Periodically diameter-modulated SiNW arrays synthesized via a Bosch-type deep reactive ion etching (DRIE) process by tuning polymer passivation and silicon etching periods. Adapted with permission from ref (25). Copyright 2016 Wiley-VCH GmbH. (d–f) Metal-assisted chemical etching (MACE) to structure Si. (d) Schematic of MACE using a metal catalyst film (in yellow) to etch through the Si (gray) showing the localized oxidation of Si in contact with the metal film due to the reduction of H_2_O_2_ at the metal film. (e) Synthesis of SiNW arrays via MACE and colloidal lithography. First row: Scheme showing the main synthesis steps. Left: a PS monolayer (in blue) is deposited on the surface via self-assembly. Center: Size reduction of the PS spheres to form a non-close-packed monolayer (not shown), followed by the deposition of a gold film and subsequent removal of the templating spheres yields a gold nanohole array. Right: MACE yields well-defined silicon nanowire arrays. Second row: Photographs of 2 in. Si wafers during the process. Third row: Corresponding secondary electron SEM images. (d, e) Adapted from ref (26). Copyright 2020 American Chemical Society. (f) Zigzag SiNW arrays fabricated via alternating MACE steps with different etchant compositions featuring different physical properties such as viscosity and surface tension. Adapted from ref (29). Copyright 2017 American Chemical Society.

A major challenge in the synthesis of SiNW arrays is the independent control of nanowire shape, dimension, and composition, while maintaining simple, fast, and large-scale fabrication capabilities. Often, exotic nanowire structures have a more complex behavior and provide additional possibilities to tailor and control functional properties. Vapor–liquid–solid (VLS) syntheses can be modified to obtain precise control over the nanowire diameter in the axial direction, by locally varying dopant concentrations (gold impurities or acceptor/donor atoms) along the lengths of the nanowire, in combination with selective chemical etching (Figure 3a,b),^22,23^ or by controlling the catalyst droplet size during the VLS growth.^24^ By controlling specific reactive ion etching (RIE) processes, nanowires can be prepared with a periodically modulated diameter^25^ (Figure 3c) or with a conical shape.^15,16^ Finally, metal-assisted chemical etching (MACE) has emerged as a low-cost, solution-based, and high-throughput technique to structure single-crystalline and polycrystalline silicon.^26−28^

#### Synthesis of SiNW Arrays via Metal-Assisted Chemical Etching (MACE) and Colloidal Lithography^26−28^

During MACE, a Si wafer covered by a nanostructured metal, usually made of a noble metal such as Au, is immersed in a solution containing hydrofluoric acid (HF) and an oxidant, typically hydrogen peroxide (H_2_O_2_) (Figure 3d).^26^ The noble metal catalyzes the reduction of the oxidant, and holes (h^+^) are formed (eq 1).
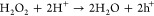
1The holes are conducted through the metal and injected into the silicon, which oxidizes and dissolves in the presence of HF, generating hydrogen gas as a byproduct (eq 2). HF is used, because it only dissolves the oxidized silicon.
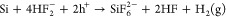
2Due to the catalytic behavior of noble metals toward the reduction of the oxidant, the redox reaction occurs much faster at the metal. As a result, the Si underneath the Au is etched preferentially. The nanostructured metal catalyst stays in intimate contact with the silicon surface thanks to attractive van der Waals interactions, leading to a homogeneous and remarkably anisotropic etching (Figure 3d).^30^ The silicon doping (type and concentration) affects both the etch rate and porosity due to band-bending that affects the hole confinement at the Au/Si interface.^28,31^ In general, MACE is independent of the crystallographic orientation.

Aside from conventional “clean room” patterning methods, colloidal lithography has appeared as a straightforward benchtop and large-scale technique to prepare the nanostructured metal film.^32,33^ It involves the deposition of a monolayer of spheres that are usually made from either polystyrene^33^ or a SiO_2_ core and a polymer shell,^34^ which form a hexagonally close-packed (hcp) array on the Si surface. The sphere monolayer is used as a template with tunable geometry and dimension for the physical deposition of the noble metal film. After lift-off, this leads to a metal nanohole array, which is then used for MACE, during which the noble metal film sinks into the silicon, producing an array of VA-SiNWs. The length of the nanowires is controlled by the MACE duration, while their diameter is controlled by the oxygen plasma duration during the size reduction of the polymeric spheres. The pitch is determined by the initial diameter of the polymeric spheres before size reduction. Thus, the combination of colloidal lithography and MACE provides a benchtop, cost-effective, and versatile way to synthesize VA-SiNW arrays with tunable geometries (Figure 3e).^26^ MACE also allows the fabrication of exotic zigzag architectures along the long-axis by adjusting the solution composition and, thus, viscosity and surface tension (Figure 3f).^29^

Achieving large-scale homogeneous SiNW arrays, such as the ones shown in Figure 3e, can be challenging because the metal catalyst might show instability during MACE when the experimental parameters are not set appropriately to (i) achieve a defect free nanostructured catalyst and (ii) ensure homogeneous adhesion of the nanostructured catalyst to the Si surface during MACE.^26^ While the colloidal mask can be fabricated by drop-casting, spin-coating, or self-assembly on the water–air interface, large-scale monolayers with only microscopic defects, such as grain boundaries, dislocations, and vacancies, are only possible using self-assembly on the water–air interface, ideally by using a Langmuir trough. Additionally, we have recently reported the critical parameters to optimize the large-scale homogeneity of the VA-SiNW array by precisely controlling etchant composition, metal film thickness, adhesion layer thickness, nanowire diameter and pitch, metal film coverage, Si/Au/etchant interface length, and crystalline quality of the colloidal template (i.e., grain size and defects).^26^ By adjusting these experimental conditions to the desired SiNW array architecture, large-scale homogeneous SiNW arrays can be fabricated with high-throughput and reproducibility.

### 3.2 Low Resolution Patterning

Many techniques are available to deposit additional materials on silicon nanowire arrays. Sputtering or thermal evaporation lead to incomplete shells or particles with more material located at the top of the SiNWs than on their sidewalls. Chemical approaches, like self-assembly, can be used to homogeneously decorate the nanowires with gold particles.^13^ Conformal metal shells can be produced via chemical vapor synthesis,^35^ electrochemical deposition,^36^ organometallic precursor pyrolysis,^37^ or wet-chemical synthesis.^38^ For the deposition of defined patterns along the nanowire axis, electron-beam lithography has been the method of choice. However, it can only be used to pattern nanostructures lying flat on a substrate and as such cannot be used to pattern three-dimensional nanoscale systems such as SiNWs arrays.^39,40^ The most promising approach to pattern specific areas of SiNW arrays is electrochemistry. By masking the bottom of the array with a photoresist layer, conformal metal shells can be electrodeposited on the top of the wires.^36^ However, synthesizing hybrid metal-SiNW arrays with exact control over the metal composition, dimension, and position along the wire axis is challenging and can only be achieved by performing three-dimensional lithography. To date, multiphoton microfabrication techniques can be used to perform lithography in three dimensions,^41^ but those are usually low-throughput, cannot produce features below 10 nm, and are limited to polymeric substrates.

## 4 Structuring and Patterning Silicon Nanowires in 3D for Spatially Controlled Light Absorption (Our Work)

**Figure 4:**
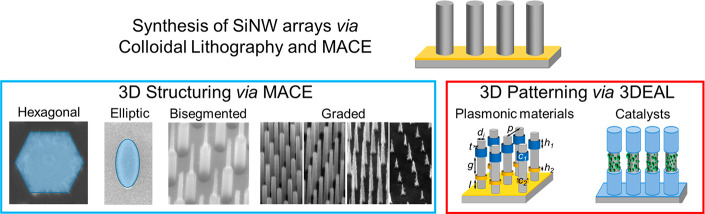
Summary of our work to structure and pattern VA-SiNW arrays in 3D via colloidal lithography, MACE, and 3DEAL. Modified from refs (11), (33), and (42) (copyright 2017, 2018, 2020, respectively, American Chemical Society), and adapted from ref (44) (2021, CC BY 3.0).

Our group has become expert in structuring and patterning SiNW arrays in three dimensions using affordable, large-scale, and solution-based chemical and electrochemical methods (Figure 4).^11,26,33,34,42−44^ The SiNW arrays are synthesized using a combination of colloidal lithography and metal-assisted chemical etching (MACE). Our benchtop approaches allow synthesizing exotic SiNW morphologies with elliptical or hexagonal cross-sections, bisegmented nanowires by combining MACE with KOH etching, substrates with graded optical properties using a dip-etching technique, and Si nanowires functionalized with plasmonic or catalytic nanostructures located at well-defined locations along the nanowires using the three-dimensional electrochemical lithography (3DEAL). Such control over the nanostructured silicon geometry provides the opportunity to (i) control light absorption within specific locations along the nanowires long-axis;^11^ (ii) produce substrates with graded morphologies and, thus, graded optical properties at the macroscale;^11^ (iii) concentrate light of desired wavelengths at specific locations by placing well-defined plasmonic structures along the nanowires, which provides homogeneous three-dimensional E-field enhancements;^42^ and (iv) define catalytically active and passive regions on SiNWs for photoelectrochemical applications.^43^

### 4.1 Controlled Nanowire Cross-Sections via Colloidal Lithography^33,44^

**Figure 5:**
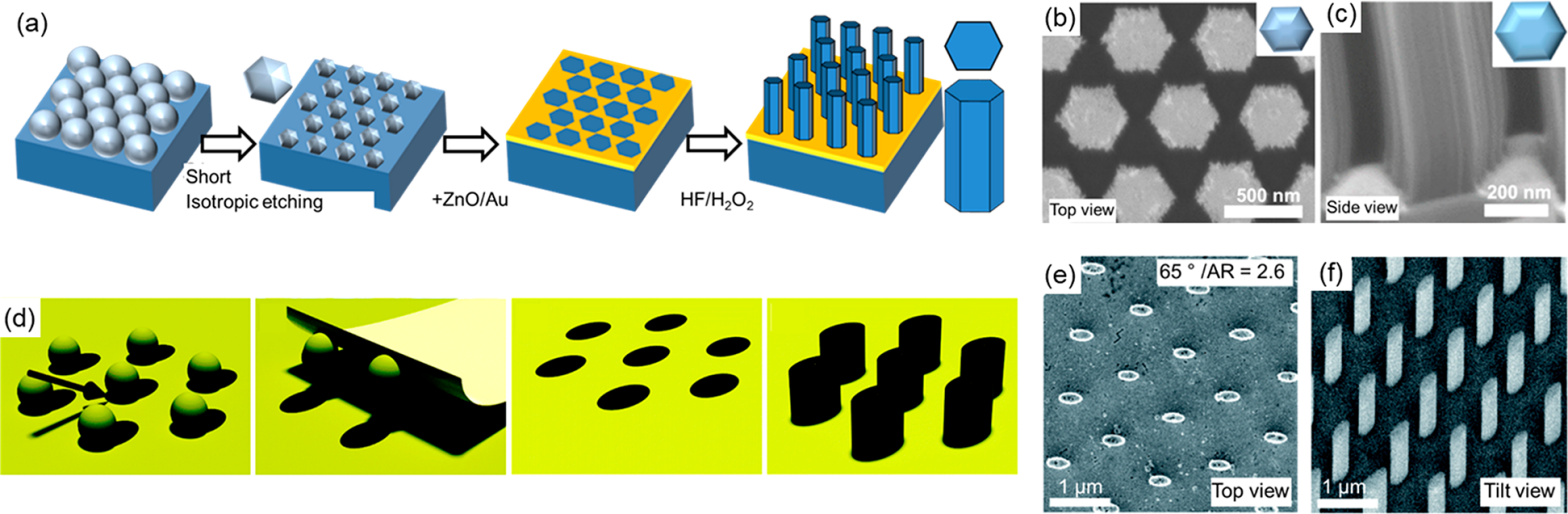
Modified colloidal lithography to control SiNW cross-section. (a–c) Hexagonal cross-sections: a short isotropic oxygen plasma etching step yields a hexagonal sphere template, which, after subsequent metal deposition and MACE, leads to hexagonal SiNWs. Adapted from ref (33). Copyright 2017 American Chemical Society. (d–f) Elliptical cross-sections: Deposition of the metal layer under an oblique angle results in an elliptical shadowing effect, leading to SiNWs with controllable elliptical morphologies after MACE. Adapted from ref (44) (2021, CC BY 3.0). Schemes (a, d) and secondary electron SEM images (b, c, e, f).

Conventional colloidal lithography is constrained by the spherical shape of the template which leads to the synthesis of SiNWs with a cylindrical morphology after MACE. Since the optical properties of SiNWs are highly dependent on their morphology,^11,45^ this clearly limits the potential of the approach. Interestingly, we found that, during a short isotropic plasma etching, the hcp PS spheres show a reduced etching rate at the contact points between the spheres, which leads to a hexagonal sphere template (Figure 5a–c). We demonstrated that such modified templates can be used to prepare SiNWs with a well-defined hexagonal morphology after MACE. By using different etching durations, it is possible to continuously adjust the wire cross-section from a perfect hexagon to a perfect circle.^33^

Instead of modifying the colloidal template morphology, it is also possible to control the SiNW morphology by using an oblique metal deposition that yields elliptical holes due to a shadowing effect (Figure 5d–f).^44^ After MACE, this yields SiNWs that have elliptical cross-sections, with aspect ratios up to 2.6 controlled by the deposition angle.

### 4.2 Synthesis of Bisegmented Nanowire Arrays^11^

**Figure 6:**
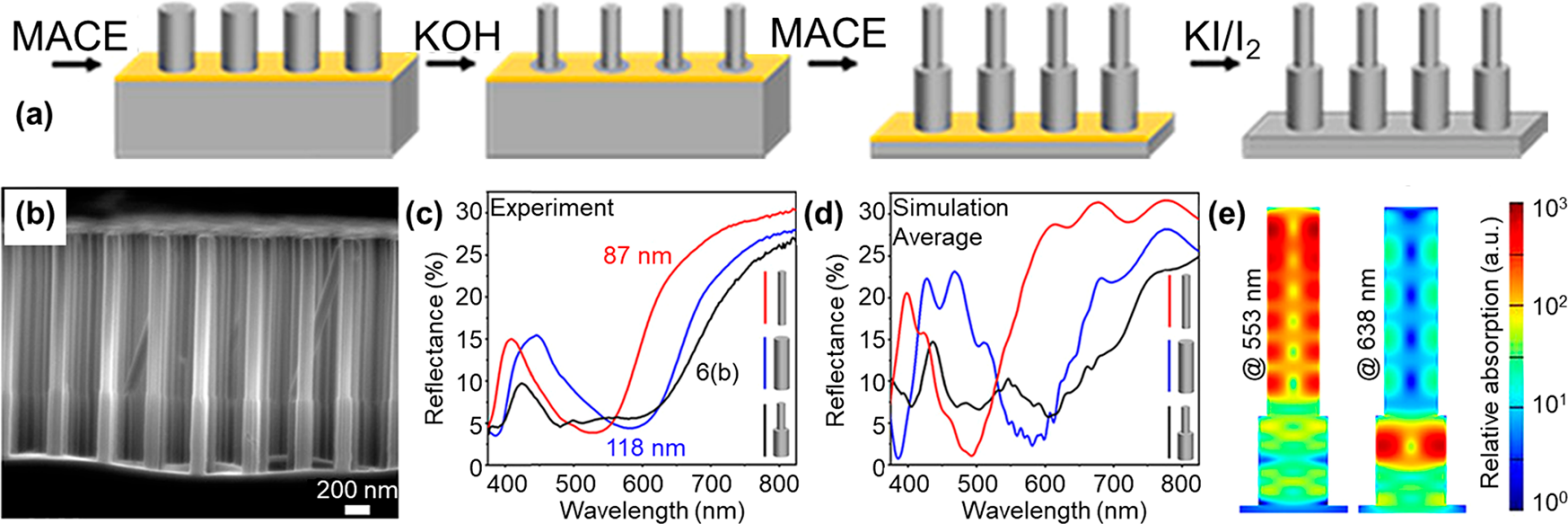
Bisegmented VA-SiNW arrays. (a) Schematic representation of the synthesis route using sequential MACE and KOH etching steps. (b) Secondary electron SEM image of bisegmented VA-SiNWs with top and bottom diameters of *d*_bi,top_ = 98 nm and *d*_bi,bottom_ = 133 nm, respectively. (c) Measured reflectance spectra of two different VA-SiNWs with a single-diameter of *d*_single1_ = 87 nm (red curve) and *d*_single2_ = 118 nm (blue curve). The black curve shows the measured reflectance of the bisegmented structure shown in panel b. (d) Simulated reflectance spectra corresponding to the VA-SiNWs shown in panel c, same color code. (e) Two-dimensional maps of the simulated relative absorption within one SiNW in the bisegmented VA-SiNW array simulated at 553 and 638 nm, with similar dimensions to the wires shown in panel b. Adapted from ref (11). Copyright 2020 American Chemical Society.

Our group recently reported the synthesis of asymmetric bisegmented VA-SiNWs via sequential MACE and KOH etching steps (Figure 6a,b).^11^ In a first step, VA-SiNWs are prepared via MACE. Next, the VA-SiNW substrate is immersed into an aqueous KOH solution that etches Si and reduces the SiNW diameter. The SiNW diameter after KOH etching can be controlled from a few tens to hundreds of nanometers by adjusting the etching time and KOH concentration. The Au nanohole film used for MACE is present at the bottom of the nanowires and is left untouched during KOH etching. As such, it protects the Si substrate and effectively directs KOH etching at the nanowires. A subsequent MACE step yields a bisegmented wire where the bottom half has a diameter inherited from the colloidal template, while the top half has a dimeter that depends on the KOH etching conditions. The length of each segment is controlled by the respective MACE durations. This procedure allows precise control over the NW diameter along the NW length. Asymmetric bisegmented VA-SiNW arrays have enhanced and tunable optical properties as demonstrated by UV–vis reflectance measurements and electromagnetic simulations using the finite-difference time-domain (FDTD) method. Figure 6c,d compares the experimental and simulated spectra of bisegmented nanowires (black curve) with the corresponding single-diameter VA-SiNWs (red and blue curves). While the single-diameter NWs show only one dip in their reflectance spectrum, corresponding to one guided mode, the bisegmented structure shows a significantly broadened reflectance dip. Absorption maps (Figure 6e) show that the smaller diameter top segment absorbs much more in the lower-wavelength range, while the larger diameter bottom segment is responsible for absorption at higher wavelengths. Overall, this leads to the observed broadening of the reflectance dip. While conventional SiNWs increase the light absorption by ∼270% in the wavelength range 375–825 nm compared to the same volume of bulk silicon, the bisegmented NWs provide an even higher enhancement of ∼300%. These results demonstrate the potential of such bisegmented VA-SiNWs to (i) increase the overall light absorption thanks to a broadening of the range of wavelengths absorbed, controlled by a precise engineering of the geometrical parameters, and (ii) spatially control light absorption at different wavelengths. The reduced reflectance and increased absorption over a broad range of wavelengths make these structures promising candidates for photovoltaic applications.^25^

### 4.3 Graded Arrays via Dip-Etching^11^

**Figure 7:**
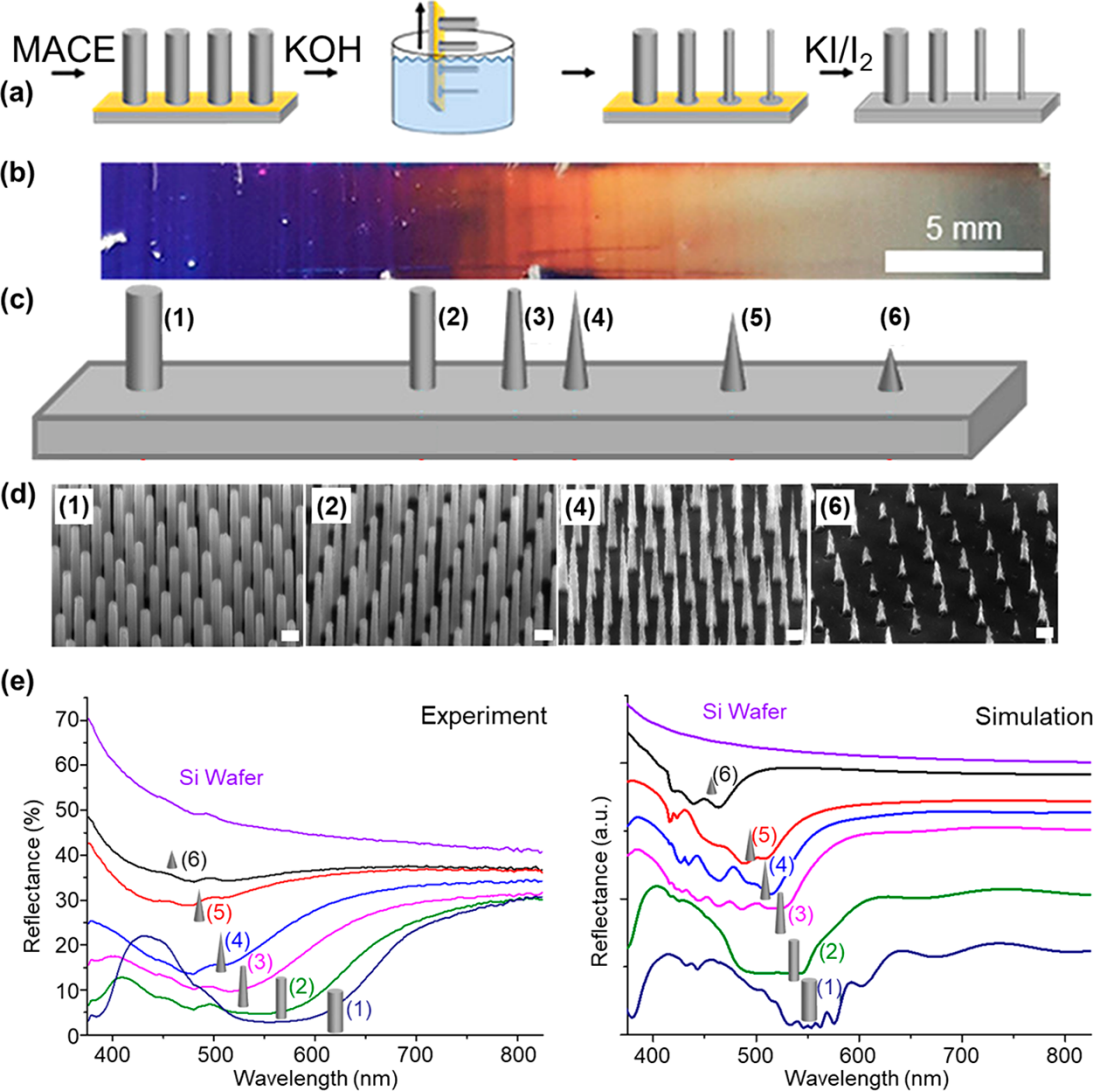
Dip-etching. (a) Schematic overview of the dip-etching process: VA-SiNWs produced via MACE are incubated in a two-phase system of aqueous KOH solution and an organic *n*-hexane phase floating on top of the KOH. Slow removal from the solution leads to varying etching times across the substrate. The Au nanohole film is dissolved in KI/I_2_ solution leading to substrates with gradually changing VA-SiNW dimensions and shapes. (b) Photograph of a gradient VA-SiNW sample (shortest KOH exposure on the left side). (c) Scheme of the gradient morphology and (d) secondary electron SEM images (scale bars: 200 nm) of selected positions indicated by the numbers 1–6. (e) Measured (left) and simulated (right) reflectance spectra for different positions on the gradient VA-SiNW structure, same positions as indicated in panel c. Adapted from ref (11). Copyright 2020 American Chemical Society.

In addition to controlling light absorption at the nanoscale, our group developed a simple dip-etching method to synthesize arrays exhibiting a shape and diameter gradient at the centimeter-scale, yielding substrates with graded optical properties (Figure 7).^11^Figure 7a shows the dip-etching principle. After the production of VA-SiNWs with a desired diameter, length, and pitch via MACE, the substrates are immersed in a two-phase mixture composed of an aqueous KOH solution at the bottom and an organic *n*-hexane phase at the top. Progressive removal of the substrate from the KOH solution leads to a substrate with gradual changes in the KOH etching duration. The organic phase is used to reduce the residual KOH adhering to the VA-SiNW substrate and obtain a more homogeneous etching.

SEM analysis demonstrates a gradual change in shape and dimensions of the SiNWs across the substrate (Figure 7b−d). Regions exposed for a short time to the KOH solution are composed of cylindrical SiNWs. The SiNW diameter gradually decreases across the substrate, while at some point the SiNWs become conical. Finally, for the longest etching times in KOH, the nanowires convert into short nanocones. The gradual change in the optical properties of these substrates is obvious by the nice progressive change in color from blue to orange to gray, seen in Figure 7b. This is also evidenced by the progressive changes seen in both the measured and simulated UV–vis reflectance spectra (Figure 7e). This approach can be used to study the influence of SiNW shape and dimensions on specific properties or functions using only one substrate.

### 4.4 Three-Dimensional Electrochemical Lithography (3DEAL)

Plasmonic materials have a strong and tunable interaction with light, which has been investigated by different scientific communities.^46−53^ From a photovoltaic point of view, plasmonic nanostructures can increase light absorption in the semiconductor by enhancing the local electric field at the localized surface plasmon resonance (LSPR) and via the direct injection of hot charge carriers generated in the plasmonic nanostructure into the semiconductor.^48,50,54^ The integration of metal nanostructures within SiNW arrays could thus be used to precisely define plasmonic hot spots in three dimensions within the array.^37,55,56^ In this regard, control over the metal nanoparticle geometrical parameters is crucial to engineer the LSPR for specific applications, as the LSPR depends on the metal, the nanoparticle size and shape, and the relative position of the nanoparticles to each other. However, the fabrication of hybrid single-crystalline Si (c-Si) nanowire architectures with defined metal nanostructures is a challenging task.

**Figure 8:**
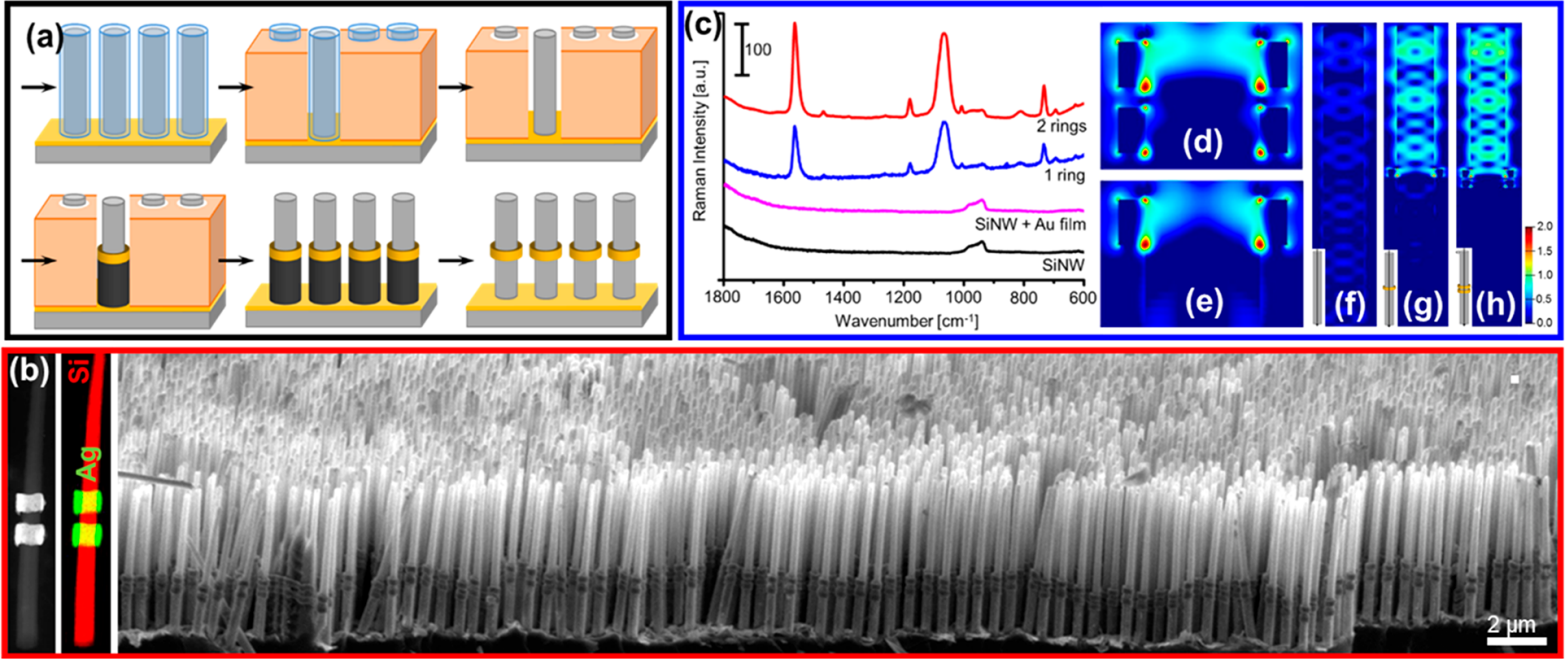
Three-dimensional electrochemical axial lithography (3DEAL) to decorate VA-SiNW with plasmonic materials. (a) Scheme showing the successive synthesis steps. After MACE, the SiNWs (in gray) are coated with a SiO_2_ shell (in blue) and are embedded within a polymer film (orange). The dissolution of the SiO_2_ shell generates annular pores. The pores guide the electrodeposition of multisegmented shells around the SiNWs starting from the gold film at the bottom (here: nickel shown in black and gold in yellow). Dissolution of the polymeric membrane and selective etching of the sacrificial shell (here: nickel) leads to a well-defined metal shell (here: a gold ring). (b) VA-SiNWs decorated with a Ag ring dimer. Left: Dark-field STEM image and corresponding EDX image (green, Ag; red, Si). Right: Low magnification of a cross-sectional secondary electron SEM image of the corresponding VA-SiNW array showing the high homogeneity of the technique. (c–h) VA-SiNW arrays patterned with Au rings. (c) Typical raw Raman spectra after 1,4-benzenedithiol functionalization of SiNWs (black curve), native SiNWs with a gold film at the bottom (magenta curve), a single Au ring array (blue curve), and a 30 nm gap ring dimer array (red curve). The spectra are offset for clarity. (d–h) E-field maps. (d, e) The SiNW region close to the Au rings is shown. (f–h) The whole nanowire is shown. (d, h) Two Au rings around a SiNW. (e, g) One Au ring around a SiNW. (f) Bare SiNW. Adapted from ref (42). Copyright 2018 American Chemical Society.

We recently developed a benchtop method, termed three-dimensional electrochemical axial lithography (3DEAL), which allows the patterning of vertically aligned crystalline silicon micro- and nanowire arrays with tailored metal architectures (Figure 8).^42^ The method is adapted from coaxial lithography (COAL), a powerful method for synthesizing metal shells around semiconductor nanowires grown from solution^57−59^ and, as such, not compatible with c-Si. Both COAL and 3DEAL are based on the sequential electrodeposition of sacrificial and target shells, which, in combination with selective etching, yields well-defined metal rings around nanowires. However, 3DEAL can be used to modify large-scale pre-existing nanostructures, such as c-SiNW arrays. In short, 3DEAL is made possible by the synthesis of a porous template with tunable dimensions that guides the electrodeposition of conformal metallic shells, which grow from the MACE base gold layer present at the bottom of the wires (Figure 8a). In the simplest case, a NiAu multisegmented shell is grown, which, after the selective etching of the Ni shells, yields isolated Au nanorings with well-defined height and position along the NW length, both determined by the charge used during the electrodeposition of the Au and Ni shells, respectively. 3DEAL is highly versatile and can be used to synthesize a variety of metal ring architectures composed of different metals (Au, Ag, Fe, and Ni) with controlled height, thickness, and position along either straight or kinked wire arrays (Figure 8b). Positive (nanorings) and negative (gaps) features are electrochemically controlled, and as such can be adjusted down to 40 and 5 nm, respectively, with relative size distributions in the 10–20% range, which are on par with state-of-the-art electrochemical templated syntheses of multisegmented nanowires.^60−62^ Additionally, the thickness of the metal shells is chemically controlled and can be adjusted in the 30–150 nm range. The technique is compatible with a wide range of array geometries: To date, we have successfully patterned VA-SiNW arrays with wire diameters and array pitches ranging from 150 nm to 1 μm and 500 nm to 1.5 μm, respectively.

3DEAL is well-suited to synthesize optically active metal nanostructures within VA-SiNW arrays that provide large E-field enhancements, as verified via Raman spectroscopy and FDTD simulations (Figure 8c–e). Indeed, functionalized SiNW arrays patterned with gold rings showed a large Raman signal compared to the reference bare SiNW arrays. Additionally, arrays with a Au ring dimer (30 nm gap) showed a higher Raman signal than the array with only one Au ring, which confirmed the E-field enhancement values expected from our FDTD simulations. Furthermore, Raman map analysis over 900 different points showed that the Raman signal and thus the E-field enhancement was highly homogeneous, with a relative standard deviation of 13%.

The FDTD simulations also indicate that the Au rings concentrate the light within specific regions of the SiNWs (Figure 8f–h): The E-field intensity in the Si located above the rings (top 1.5 μm of the SiNW) is enhanced by a factor of ∼2 for a single Au ring (Figure 8g) and ∼3 for a Au ring dimer (Figure 8h) compared to a pristine Si nanowire (Figure 8f), while it is nearly suppressed below the rings (bottom 1.5 μm of the SiNW). These results together with the remarkably small standard deviation obtained from Raman measurements demonstrate the reliability and potential of the method to engineer plasmonic fields and to spatially control light absorption within SiNW arrays.

Furthermore, the enhanced optical properties of silicon–metal hybrids have rendered them great platforms for photoelectrochemical studies.^17,19,37,63^ In this context, the ability to control the precise location of catalytic metals on the nanostructured silicon provides the possibility to optimize catalyst loading, improve conversion efficiencies, and lower fabrication costs.^43,64^ Indeed, the local catalyst activity can be affected by inhomogeneous mass-transport, charge recombination, light absorption, catalyst coverage, and defect distribution,^21,65,66^ which can be mitigated by optimizing the catalyst location at the semiconductor surface.^64,67^ Nanostructured silicon is a favorable photocathode for solar water splitting, thanks to the high abundance of Si, highly defined and tunable electronic properties, appropriate band gap for solar light absorption, and proper band-edge energy for the hydrogen evolution reaction (HER).^17^ Because of the slow reduction kinetics of protons, decorating silicon photoelectrodes with catalysts is necessary to achieve high conversion efficiencies. However, the catalyst position within the SiNW arrays can play a decisive role for the conversion efficiencies, as discussed above. The possibility to precisely engineer hybrid metal-SiNW architectures could help to further understand the influence of catalyst location and loading on parasitic light absorption in the metal catalysts, local light absorption in the semiconductor, relative values of the minority charge carrier-diffusion length, and local mass-transport to the catalyst.

**Figure 9:**
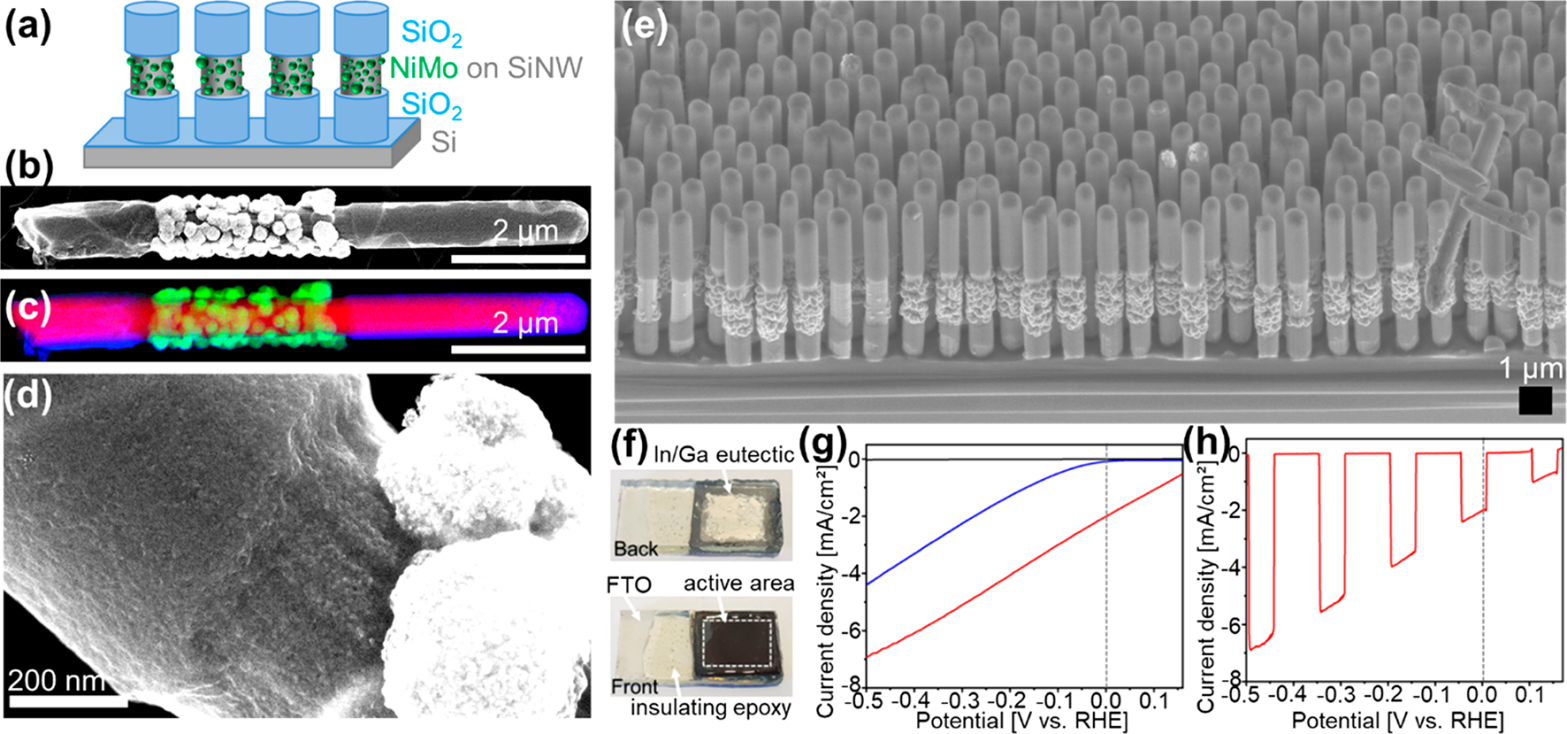
3DEAL for the spatioselective deposition of catalytic materials. (a) Scheme showing the final structure. (b–h) SiNWs patterned with SiO_2_ and a Ni–Mo catalyst. (b) Secondary electron STEM image. (c) STEM elemental map (red, Si; blue, O; green, Ni). (d) High-magnification secondary electron STEM image. (e) Secondary electron SEM image. Tilt angle: 30°. Image width: 30 μm. (f) Photograph of a typical photocathode. Top: Back side. Bottom: Front side. (g, h) Linear sweep voltammograms for the hydrogen evolution reaction in 0.1 M H_2_SO_4_, pH = 1. Light source: Calibrated solar simulator with an AM1.5G filter to a light irradiance of *P* = 120 mW/cm^2^. The potentials are all given versus the reversible hydrogen electrode potential (RHE). The vertical gray dotted lines are guide to the eyes to show the 0 V vs RHE potential. (g) Blue curve: Bare SiNW arrays. Red curve: SiNW array patterned with Ni–Mo catalysts. Black curve: Dark current. (h) Chopped illumination. Adapted from ref (43). Copyright 2020 American Chemical Society.

To provide the community with a synthetic platform affording such complex studies, we modified the original 3DEAL to pattern SiNW arrays with insulating SiO_2_ shells, which direct the electrochemical deposition directly at the exposed regions on the SiNW surface (Figure 9). Because the high surface area of nanostructured photoelectrodes can lead to an increased recombination rate and decreased open-circuit potential, the ability to passivate specific regions of the SiNWs should lead to higher efficiencies.^17,19−21^

We prepared VA-SiNW photocathodes patterned with insulating SiO_2_ shells and a Ni–Mo alloy catalyst (Figure 9a–f) and tested them for the hydrogen evolution reaction (HER). Linear sweep voltammetry in 0.1 M H_2_SO_4_ (pH = 1) under light irradiation (300 W Xe lamp equipped with an AM1.5G filter calibrated to 120 mW/cm^2^) demonstrates the viability to pattern SiNW arrays with functional metal electrocatalysts (Figure 9g,h). The onset potential, at which the current reaches 1 mA/cm^2^, increases from −0.17 V (vs reversible hydrogen electrode, RHE) for the bare wires (Figure 9g, blue curve) to +0.11 V (vs RHE) for the SiNW array partially coated with Ni–Mo catalysts (red curve). This demonstrates the potential of the technique to investigate the influence of catalyst location on the conversion efficiencies of nanostructured photoelectrodes.

## 5 Conclusions

Thanks to their fascinating and tunable optical properties, VA-SiNW arrays provide exciting opportunities for photovoltaics, photodetection, photoelectrochemistry, and sensing. The methods described here can be used to perform three-dimensional, high-resolution structuring and patterning of VA-SiNW arrays, which provide additional degrees of freedom to manipulate light at both the nanoscale and the macroscale. The 3DEAL is a powerful technique for decorating VA-SiNW arrays with optically and catalytically active nanomaterials and, as such, should be relevant to a variety of research areas. In particular, 3DEAL has the potential to tackle important challenges in photoelectrochemical devices, such as optimization of catalyst loading, investigation of charge separation and transfer within nanostructured photoelectrodes, and further improvement of metal/semiconductor photoelectrode conversion efficiencies. Because our synthetic approach involves simple benchtop and chemical techniques that are available to most researchers, it should benefit most groups interested in and working on nanostructured silicon substrates.

## Abbreviations

*d*: diameter
*l*: length
*p*: pitch (nanowire center-to-center distance)
b-Si: black silicon
c-Si: crystalline silicon
COAL: coaxial lithography
3DEAL: three-dimensional electrochemical axial lithography
FDTD: finite-difference time-domain
LSPR: localized surface plasmon resonance
MACE: metal-assisted chemical etching
PS: polystyrene
RIE: reactive ion etching
RHE: reversible hydrogen electrode
VA-SiNW: vertically aligned silicon nanowires
VLS: vapor–liquid–solid
